# Sleep oscillation-specific associations with Alzheimer’s disease CSF biomarkers: novel roles for sleep spindles and tau

**DOI:** 10.1186/s13024-019-0309-5

**Published:** 2019-02-21

**Authors:** Korey Kam, Ankit Parekh, Ram A. Sharma, Andreia Andrade, Monica Lewin, Bresne Castillo, Omonigho M. Bubu, Nicholas J. Chua, Margo D. Miller, Anna E. Mullins, Lidia Glodzik, Lisa Mosconi, Nadia Gosselin, Kulkarni Prathamesh, Zhe Chen, Kaj Blennow, Henrik Zetterberg, Nisha Bagchi, Bianca Cavedoni, David M. Rapoport, Indu Ayappa, Mony J. de Leon, Eva Petkova, Andrew W. Varga, Ricardo S. Osorio

**Affiliations:** 1 Mount Sinai Integrative Sleep Center, Division of Pulmonary, Critical Care, and Sleep Medicine, Icahn School of Medicine at Mount Sinai, One Gustave L. Levy Place, Box 1232, New York, NY 10029 USA; 2 Department of Psychiatry, NYU School of Medicine, New York, NY 10016 USA; 3 Nathan Kline Institute for Psychiatric Research, Orangeburg, NY 10962 USA; 4 Department of Neurology, Weill Cornell Medical College, New York, NY USA; 5 Center for Advanced Research in Sleep Medicine (CARSM), Department of Psychology, Hospital du Sacré-Coeur de Montreal, Montreal, Quebec, Canada and Université de Montreal, Montreal, Quebec Canada; 6 Institute of Neuroscience and Psychiatry, Department of Psychiatry and Neurochemistry, the Sahlgrenska Academy at the University of Gothenburg, Mölndal, Sweden; 7 Clinical Neurochemistry Laboratory, Sahlgrenska University Hospital, Mölndal, Sweden; 8 Department of Molecular Neuroscience, UCL Institute of Neurology, Queen Square, London, UK; 9 UK Dementia Research Institute at UCL, London, UK; 10 Child and Adolescent Psychiatry, NYU School of Medicine, New York, NY 10016 USA

## Abstract

**Background:**

Based on associations between sleep spindles, cognition, and sleep-dependent memory processing, here we evaluated potential relationships between levels of CSF Aβ_42_, P-tau, and T-tau with sleep spindle density and other biophysical properties of sleep spindles in a sample of cognitively normal elderly individuals.

**Methods:**

One-night in-lab nocturnal polysomnography (NPSG) and morning to early afternoon CSF collection were performed to measure CSF Aβ_42_, P-tau and T-tau. Seven days of actigraphy were collected to assess habitual total sleep time.

**Results:**

Spindle density during NREM stage 2 (N2) sleep was negatively correlated with CSF Aβ_42_, P-tau and T-tau. From the three, CSF T-tau was the most significantly associated with spindle density, after adjusting for age, sex and ApoE4. Spindle duration, count and fast spindle density were also negatively correlated with T-tau levels. Sleep duration and other measures of sleep quality were not correlated with spindle characteristics and did not modify the associations between sleep spindle characteristics and the CSF biomarkers of AD.

**Conclusions:**

Reduced spindles during N2 sleep may represent an early dysfunction related to tau, possibly reflecting axonal damage or altered neuronal tau secretion, rendering it a potentially novel biomarker for early neuronal dysfunction. Given their putative role in memory consolidation and neuroplasticity, sleep spindles may represent a mechanism by which tau impairs memory consolidation, as well as a possible target for therapeutic interventions in cognitive decline.

**Electronic supplementary material:**

The online version of this article (10.1186/s13024-019-0309-5) contains supplementary material, which is available to authorized users.

## Background

With 10% of adults over the age of 65 suffering from dementia [1, 2] and this number projected to double by 2050 [3], understanding the factors responsible for cognitive impairment is of critical importance [4]. Amyloid beta (Aβ) plaques and neurofibrillary tangles (NFTs) are two key pathological processes that are thought to lead to cognitive deterioration in Alzheimer’s disease (AD). Cerebrospinal fluid (CSF) Aβ and tau concentrations have been used extensively as biomarkers of AD pathology and found to correlate with plaques and tangles at post-mortem [5–8]. Emerging evidence suggests that the sleep-wake cycle directly influences their levels in older adults [9, 10]. Specifically, low NREM stage 3 (N3) slow wave activity (SWA) is associated, in healthy midlife and young-old, with trait-markers of high cerebrospinal fluid (CSF) Aβ [11, 12], while active disruption of slow wave sleep (SWS) results in state-dependent CSF Aβ peptide increases within subjects [13]. Sleep disruption has also been associated with increased tau pathology in both animal models [14–17] and humans [18], but the precise mechanisms are not known. Unlike Aβ, associations between poor SWS and CSF tau have not been reported [12, 13], suggesting that disruption in other sleep oscillations could be linked to tau pathology.

Cortical sleep spindles are 11–16 Hz bursts of activity generated within the thalamo-cortical network that occur during N2–3 and have been defined as slow or fast based on their spectral frequency [19]. A decrease in sleep spindle activity is a good candidate to be associated with tau pathology for several reasons. First, early structures affected by NFTs include sleep/wake regulating centers such as the locus coeruleus [20], suggesting that changes to sleep architecture might be one of the early outward manifestations of tau pathology. Second, sleep spindles are known to decline with age [21, 22], with a specific decline in fast spindles in mild cognitive impairment (MCI) and AD that predicts low MMSE scores [23]. Third, sleep spindles are also associated with sleep-dependent improvement in motor learning [24–27] and memory [28–34], with differential roles for fast and slow spindles depending on age [35–37]. A decrease in these neuroplasticity-promoting processes could render a greater vulnerability to the spread of tau pathology. Therefore, the mutual associations of cognition with both tau and sleep spindles raises the possibility of ties between them, particularly in older adults. Indeed, NFT load corresponds more closely to cognitive status than Aβ plaques [38], and both AD and MCI patients show significant spindle density reductions [23]. In this study, we sought to determine the relationship between sleep spindle activity in N2 and CSF biomarkers in a population of cognitively normal older adults. We carried out additional analyses looking into other spindle biophysical properties such as spindle count, duration, peak frequency, and slow/fast spindle density. We also explored whether sleep quality measured with polysomnography or habitual sleep duration measured with actigraphy is associated with CSF AD biomarkers.

## Methods

### Participants and clinical evaluation

Fifty subjects were recruited from a pool of healthy elderly participating in NIH-supported longitudinal studies on normal aging and biomarkers of AD at NYU. All subjects had ≥12 years of education, received the standardized Uniform Data Set II diagnostic assessment [39], and were non-depressed (as defined by Geriatric Depression Scale < 6), cognitively normal and in good overall health. Cognitive tests included to measure declarative memory were subtests of the Guild Memory Scale: verbal paired associates, delayed paragraph recall subtest and the Wechsler Memory Scale Revised: Logical Memory subtests (Logic I and II). A subtest of the Wechsler Intelligence Scale Revised was added to assess working memory (digits backward). The Digit Symbol Substitution Test (DSST) was used to evaluate psychomotor speed. Trails A and digits forward were included to evaluate attention and Trails B Test was included to evaluate executive function. Category fluency (animals and vegetables) and the Boston Naming Test were used to evaluate language. The Mini Mental State Examination was included as an additional global measure of cognition. Cognitive performance data were normalized using z-scores adjusting for age, sex, race, and years of education as previously reported [40]. In addition, all subjects had clinical labs to fulfill eligibility criteria and underwent structural brain MRI. Individuals with documented obstructive sleep apnea (OSA), defined as an Apnea Hypopnea Index with 4% Desaturation [AHI4%] ≥15 per hour, active continuous positive airway pressure (CPAP) use, or history of significant conditions that may affect brain structure or function such as stroke, uncontrolled diabetes, traumatic brain injury, lung diseases, drug abuse or MRI evidence of intracranial mass or infarcts were excluded. Both the NYU and ISMMS institutional review boards approved the inclusion of human participants for this study. All participants provided written informed consent.

### Lumbar puncture, CSF collection and analysis

Procedures for the lumbar punctures (LP) performed at NYU have been previously published [41, 42]. Briefly, all LPs were performed in the morning to early afternoon (average time of LP was 12:17 pm ± 57 min, and average duration between awakening time and LP was 5 h and 27 ± 54 min). The mean interval between the nocturnal polysomnography (NPSG) and CSF collection was 4.9 ± 6.8 months. Concentrations of CSF total-tau (T-tau), tau phosphorylated at threonine 181 (P-tau) and amyloid beta 42 (Aβ_42_) were measured using enzyme-linked immunosorbent assays (*INNOTEST, Fujirebio, Belgium*) conducted at the Sahlgrenska University Hospital (Sweden) blind to both clinical and sleep data.

### In-lab nocturnal polysomnography (NPSG)

Sleep recordings were performed following American Academy of Sleep Medicine (AASM) guidelines [43]. Briefly, NPSG consisted of six electroencephalographic (EEG) channels (F3–4, C3–4 and O1–2 referenced to the contralateral mastoid), two electrooculographic (EOG) leads, and one chin electromyographic (EMG) channel. Visual scoring of sleep/wake stages into 30-s epochs of wakefulness (W), NREM 1 sleep (N1), NREM 2 sleep (N2), NREM 3 sleep (N3), REM sleep (R) was performed by a single scorer blind to CSF data [12]. Sleep quality measures including total sleep time (TST), wake after sleep onset (WASO), sleep efficiency (SE), arousals and respiratory events were scored using AASM criteria [43]. AHI4% was defined as the sum of all apneas and hypopneas with ≥4% desaturation divided by TST in hours. AHI-all was defined as the sum of all apneas and hypopneas with ≥3% desaturation or arousal divided by TST in hours. Characteristics of the sleep spindles were obtained from the EEG signals, acquired with a sampling frequency of 256 Hz. Based on the optimal identification of spindles [44–46], the C3-lead was chosen for spindle detection. We used our published DETOKS [44] method to decompose the EEG channel into oscillatory and non-oscillatory or transient components. Spindles with duration less than 0.5 and more than 3 s were discarded. Detected spindles were further classified as either fast (13–16 Hz) or slow (11–13 Hz) based on their power spectra, calculated using the fast Fourier transform (FFT). Absolute SWA was calculated using the average power density by FFT in the 0.5–4.0 Hz range at the F4 EEG lead [47].

### Habitual sleep assessment

Wrist-based actigraph (Micro Motionlogger, AMI Inc.) data was collected to measure at home objective measures of TST. Subjects wore the actigraph for seven consecutive days on the non-dominant hand and maintained sleep logs to help confirm actigraphy measures.

### Statistical analyses

CSF and spindle properties were not normally distributed (Shapiro-Wilk test), therefore Spearman correlations were performed for bivariate analysis. Although correlations do not imply directionality, in order to better understand how individual CSF AD biomarkers could be influencing spindle density, we performed linear regression analyses where spindle density was the response variable. To restore normality for regression analyses, a square root transformation was applied to spindle density in N2, spindle count in N2 and fast spindle density in N2, while natural log transformations were applied to WASO, AHI4%, AHI-all, arousal index and CSF variables. Box-Cox transformation was applied to spindle duration and SE. Before conducting the regression analyses, we checked for inter-correlations between all the above variables. A correlation matrix was computed on transformed variables using Pearson’s correlations. Variables that inter-correlated with a value higher than 0.6 (i.e. P-tau and T-tau: r = 0.929, *p* < 0.001; WASO and SE: r = 0.931, p < 0.001; AHI4% and AHI-all: r = 0.709, *p* < 0.001; AHI-all and arousal index: r = 0.754, p < 0.001) were not included in the same regression models. Three predefined models of adjustment were used in which spindle density in N2 was the response variable. Model 1 included age, sex and ApoE4 status as predictors. Model 2 added each CSF biomarker individually as a predictor variable to age, sex, and ApoE4 status. Model 3 included combinations of CSF biomarkers (i.e. CSF Aβ_42_ and T-tau or Aβ_42_ and P-tau) along with age, sex, and ApoE4 status. After establishing T-tau as the strongest CSF predictor of spindle density, on a final analysis we assessed the extent to which spindle density best predicted variance in CSF T-tau (Models 4 and 5) or the CSF T-tau/Aβ_42_ ratio (Models 6 and 7) among several alternative sleep physiology variables by performing hierarchical regression with CSF T-tau or the CSF T-tau/Aβ_42_ ratio as the response variable. Models 4 and 6 included age, sex and ApoE4 status as predictors. Models 5 and 7 added individual sleep measures as predictors including N2 spindle density, frontal SWA, WASO, SE, AHI4%, AHI-all, in-lab TST, and habitual TST. All statistical analyses were performed with SPSS version 23.0 (IBM Corp., Armonk, NY). Statistical significance was set at *p* < 0.05 using two-tailed tests.

## Results

### Participant characteristics

A total of 50 subjects (54% female) with mean age 67.2 ± 7.3 (range 53–83) participated in this study. Table 1 lists demographic characteristics of this sample, including ethnicity, common medical comorbidities, and measures of cognition, sleep, and levels of T-tau, P-tau, and Aβ_42_ in the CSF. All subjects had a Clinical Dementia Rating (CDR) of 0, mean Mini-Mental State Examination (MMSE) of 29.1 ± 1.1, 16.7 ± 2.1 years of education, and were generally non-obese (BMI = 25.4 ± 3.5). Thirty-four percent (17 subjects) were ApoE4*+*. Overall, the cohort consisted of cognitively normal elderly in good health with 4 of 50 that were both amyloid-positive and tau positive (Aβ_42_ < 469.5 pg/mL, T-tau > 323 pg/mL or P-tau > 52.8 pg/mL as positive cut-offs), 14 of 50 that were amyloid-negative and tau-positive, 8 of 50 that were amyloid-positive and tau-negative, and 24 of 50 that were both amyloid-negative and tau-negative (Table 1). These cut-off values were calculated based on construction of ROC curves for healthy and diseased subjects (MCI and AD) using the NYU Center for Brain Health biobank. Histograms of the distributions of CSF Aβ_42_, T-tau, and P-tau in this cohort are shown in Additional file 1: Figure S1). In-lab TST was 6.03 ± 1.05 h with WASO of 91.5 ± 58.3 min and SE of 78.3 ± 11.9% (Table 1). This cohort did not suffer from subjective daytime sleepiness as determined by the Epworth Sleepiness Scale (ESS = 5.9 ± 3.7). In-lab sleep latency was 11.7 ± 12.7 min and latency to REM was 99.2 ± 61.9 min. Median AHI4% was 1.2 (IQR 2.9/h, range 0.1/h to 10.8/h) and median AHI-all was 8.5 (IQR 7.8/h, range 1.8/h to 29.3/h). No associations were observed between spindle density and age, likely a consequence of the relatively restricted age range of our sample. Similarly, although women generally have higher spindle density than men [21, 48], in this sample there were no significant differences in N2 spindle density across sex (males: 1.9 ± 1.3 #/min N2 sleep, females: 2.2 ± 1.5 #/min N2 sleep, *p* = 0.566).

**Table 1:** Participant characteristics

	n = 50
Age	67.2 ± 7.3
Male	46% (23)
BMI	25.4 ± 3.5
Education	16.7 ± 2.1
CDR	0
MMSE	29.1 ± 1.1
Hypertension	30% (15)
Cardiovascular disease	6% (3)
Diabetes	2% (1)
Thyroid disorders	18% (9)
ApoE4+	34% (17)
Ethnicity	
Caucasian	84% (42)
African American	14% (7)
Asian	2% (1)
CSF (pg/mL)^1^	
Aβ_42_ median (IQR)	626.0 (339)
P-tau_181_ median (IQR)	40.1 (21)
T-tau, median (IQR)	241.7 (192)
Preclinical AD groups	
Amyloid^−^/Tau^−^	24 (48%), 3 (12.5%) ApoE4 carriers
Amyloid^−^/Tau^+^	14 (28%), 6 (42.9%) ApoE4 carriers
Amyloid^+^/Tau^−^	8 (16%), 4 (50%) ApoE4 carriers
Amyloid^+^/Tau^+^	4 (8%), 4 (100%) ApoE4 carriers
Sleep	
ESS	5.9 ± 3.7
AHI4% (IQR)	1.2 (2.9)
AHI-all (IQR)	8.5 (7.8)
Arousal Index	19.7 ± 7.6
O_2_ Saturation	94.4 ± 1.7
In-lab TST (hrs.)	6.0 ± 1.0
Latency to sleep (min.)	11.7 ± 12.7
Latency to REM (min.)	99.2 ± 61.9
WASO (min.)	91.5 ± 58.3
SE (%)	78.3 ± 11.9
N1 (% of TST)	20.4 ± 8.3
N2 (% of TST)	43.6 ± 10.8
N3 (% of TST)	17.6 ± 10.8
REM (% of TST)	18.4 ± 4.9
Habitual TST (hrs.)	7.2 ± 1.0

Results reported as mean ± SD with the exception of CSF and AHI data which are reported as median (interquartile range, IQR)

1. Biomarker profile determined using Aβ_42_ cutoff < 500 pg/mL and P-tau_181_ cutoff > 52.9 pg/mL or T-tau cutoff > 323 pg/mL with percent of cohort and number of subjects per group (including number of E4 carriers and non-carriers) reported

Abbreviations: *CDR* Clinical Dementia Rating, *MMSE* Mini Mental State Examination, *CSF* cerebral spinal fluid, *ESS* Epworth Sleepiness Scale, *AHI4%* Apnea Hypopnea Index with 4% Desaturation, *AHI-all* all Apneas Hypopneas and Arousals Index, *TST* Total sleep time, *WASO* Wake after sleep onset, *SE* Sleep efficiency, N1: Stage N1 sleep, N2: Stage N2 sleep, N3: Stage N3 sleep, REM: Rapid-eye movement sleep

### Association between N2 spindle density, CSF tau proteins and Aβ_42_

Pairwise unadjusted and conditional (on age, sex and ApoE4 status) correlations between the CSF biomarkers of AD and N2 sleep spindles density are presented in Table 2. The unadjusted correlations are given below the main diagonal while conditional correlations are given above the main diagonal. There was a high inter-correlation between CSF AD biomarkers, particularly between T-tau and P-tau (r = 0.929, *p* < 0.001). N2 spindle density was negatively correlated with all CSF measures, and despite the strong correlation between both CSF tau measures, spindle density was more strongly associated with T-tau than with P-tau. The pairwise correlation coefficients adjusted for age, sex and ApoE4 status, given above the main diagonal, show a similar pattern and indicate that the conditional associations were even stronger than the unadjusted ones. In addition to individual CSF protein levels, the ratios between T-tau/Aβ_42_ and P-tau/Aβ_42_ have been used as AD biomarkers. Spindle density during N2 sleep was also found to be negatively correlated with the ratio of T-tau/Aβ_42_ (r = − 0.380, *p* = 0.010) and P-tau/Aβ_42_ (r = − 0.31, *p* = 0.043).

**Table 2:**
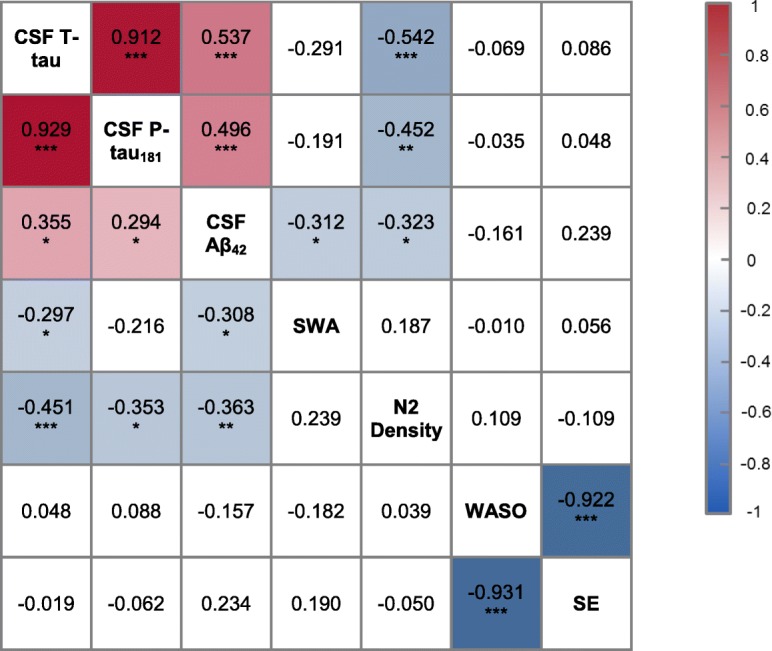
Correlation matrix of CSF proteins, SWA, N2 spindle density, and sleep quality measures

Partial Pearson correlations controlling for age, sex, and ApoE4 genotype presented above the diagonal, unadjusted Pearson correlations presented below the diagonal. Significant correlations are color coded with shade indicated by r value. * = *p* < 0.05, ** = *p* < 0.01, *** = *p* < 0.001.

To evaluate the relative strength of the association of each of the CSF biomarkers with N2 spindle density, we conducted hierarchical regression analysis with N2 spindle density as the response and each of the CSF biomarkers as the predictors. The results are given in Table 3. The model with only the age, sex, and ApoE4 genotype and no CSF predictors explained 12.8% of the variance in spindle density (Model 1). In the presence of the covariates, each of the CSF biomarkers was a significant predictor, and adding each of the variables improved the predictive power: Aβ_42_ by 8.8%, P-tau by 18.1%, T-tau by 25.9%, and the T-tau/Aβ_42_ ratio by 12.9% (see ΔR^2^ in Table 3), confirming the results discussed above. Finally, inclusion of pairs of CSF biomarkers together (Model 3) showed that in the presence of T-tau or P-tau, Aβ_42_ did not remain a significant predictor of N2 spindle density, while T-tau and P-tau remained significant predictors in the presence of Aβ_42_, albeit with T-tau having greater predictive value. There was a dramatic decrease in magnitude of the coefficients for Aβ_42_ from Model 2, where it is the single CSF measure, to Model 3, where it is together with T-tau (Table 3), while the T-Tau coefficients remained unchanged or increased in strength in the presence of Aβ_42_. This suggests the principle importance of T-tau among the three CSF biomarkers in their association with N2 spindle density.

**Table 3:** Hierarchical linear regression examining spindle density as a function of CSF proteins

Model ^a^	Predictors	β	95% CI	p ^d^	R^2^	ΔR^2^
Model 1: age, sex and ApoE4 status only	Age	−0.251	− 0.033, 0.002	0.081	0.128	NA
	Sex	0.032	−0.234, 0.294	0.819		
	APOE ε4	0.232	−0.045, 0.500	0.100		
Models 2 ^b^: age, sex and ApoE4 status plus one CSF biomarker	Aβ_42_	−0.312	−0.79, − 0.043	0.030	0.216	0.088*
	P-tau_181_	−0.456	−0.907, − 0.236	0.001	0.308	0.181*
	T-tau	−0.539	−0.783, − 0.288	< 0.001	0.386	0.259*
	T-tau/Aβ_42_ ratio	−0.415	−4.504, − 0.735	0.008	0.257	0.129*
Models 3 ^c^: age, sex and ApoE4 status plus two CSF biomarkers	T-tau	−0.503	−0.784, − 0.216	0.001	0.390	0.004
	+ Aβ_42_	−0.075	−0.479, 0.279	0.598		
	Aβ_42_	−0.152	−0.589, 0.182	0.294	0.325	−0.076*
	+ P-tau_181_	−0.391	−0.859, − 0.121	0.010		

a. dependent variable: N2 spindle density

b. change from model with covariates age, sex and ApoE4

c. change from model with covariates age, sex, ApoE4 and CSF T-tau

d. significance level for each predictor

* denotes significant change in R^2^ from comparator model (p < 0.05)

### CSF measures of tau do not correlate with measures of sleep quality or habitual sleep duration

In order to ascertain that the influence of tau is specific to spindles, we investigated potential relationships between T-tau and P-tau and sleep quality variables measured with PSG, including sleep efficiency, WASO, AHI4% and AHI-all, as well as variables measured with actigraphy, including habitual sleep duration (TST). We did not observe any correlations between T-tau or P-tau and any of these measures at baseline (Additional file 1: Figure S2).

To further confirm the specificity of sleep spindles in predicting CSF T-tau, we conducted hierarchical regression analysis with CSF T-tau as the response variable with several sleep measures as predictors (Table 4). The baseline model with age, sex, and ApoE4 genotype alone without sleep predictors explained 7.7% of the variance in CSF T-tau (Model 4, Table 4). Next, we added sleep predictors individually to assess whether or not they improved the predictive power of CSF T-tau (Model 5, Table 4). Of the sleep predictors selected, N2 spindle density significantly improved the predictive power of the model for CSF T-tau by 28.4% above age, sex, and ApoE4 genotype (ΔR^2^ = 0.284, *p* = 0.001, Model 5, Table 4), and it was the only sleep variable to do so. The addition of comorbidities including presence of hypertension, cardiovascular disease, diabetes and thyroid disorders did not influence the relationship between N2 density and CSF T-tau (Additional file 2: Table S2). There were also no significant correlations between N2 spindle density and any of the other sleep variables examined in this cohort.

**Table 4:** Hierarchical linear regression examining CSF T-tau as a function of sleep measures

Model ^a^	Predictors	β	95% CI	p ^c^	R^2^	ΔR^2^
Model 4: age, sex and ApoE4 status only	Age	0.065	−0.016, 0.024	0.713	0.077	NA
	Sex	−0.01	− 0.310, 0.297	0.966		
	ApoE4	0.262	−0.074, 0.532	0.134		
Models 5 ^b^: age, sex and ApoE4 status plus one sleep variable	N2 spindle density	−0.573	−0.772, − 0.224	0.001	0.361	0.284*
	SWA	−0.265	−0.494, 0.099	0.184	0.129	0.052
	WASO	−0.247	−0.408, 0.085	0.191	0.127	0.050
	SE	0.310	−0.110, 1.672	0.084	0.163	0.086
	AHI4%	−0.248	−0.185, 0.029	0.149	0.138	0.061
	AHI-all	−0.278	−0.404, 0.057	0.135	0.142	0.065
	TST in-lab	0.171	−0.082, 0.229	0.345	0.103	0.027
	TST actigraphy	−0.063	−0.178, 0.129	0.749	0.080	0.003

a. dependent variable: CSF T-tau

b. change from model which only includes covariates age, sex, and ApoE4

c. significance level for each predictor

* denotes significant change in R^2^ from comparator model (p < 0.05)

In a related model, we conducted hierarchical regression analysis with the CSF T-tau/Aβ_42_ ratio as the response variable with several sleep measures as predictors. ApoE4 was such a significant predictor of CSF Aβ_42_ that the combination of age, sex, and ApoE4 genotype explained 31% of the variance in the CSF T-tau/Aβ_42_ ratio. (Additional file 3: Table S1, Model 6). In this analysis, there is no sleep variable that significantly explained additional variance in CSF T-tau/Aβ_42_ ratio beyond age, sex, and ApoE4 genotype, although N2 spindle density comes closest (ΔR^2^ = 0.07, *p* = 0.072 (Additional file 3: Table S1, Model 7).

### CSF tau associates with several spindle biophysical properties, but not with SWA

**Fig. 1:**
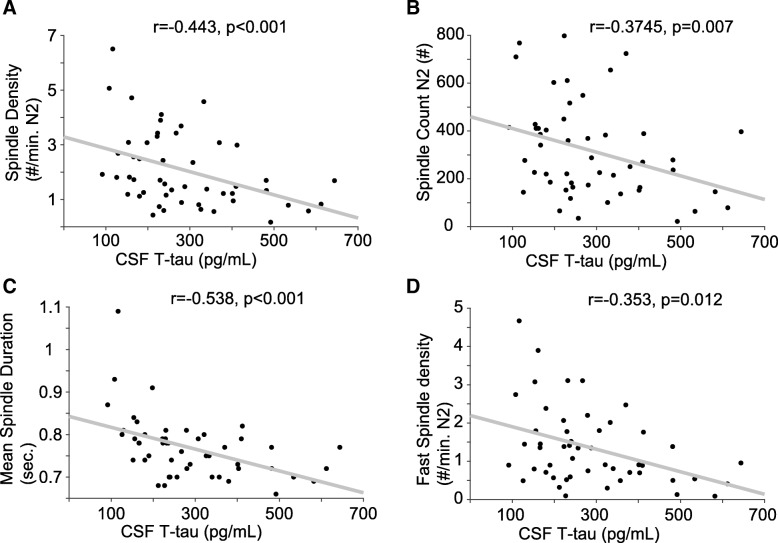
*Correlations between sleep spindle properties and CSF T-tau*. Scatter plots of N2 spindle density (#/min. N2 sleep) (**a**), N2 spindle count (**b**), spindle duration (sec.) (**c**) and N2 fast spindle density (#/min. N2 sleep) (**d**) with CSF T-tau indicate significant associations at cross-section (*n* = 50 subjects)

We observed that CSF T-tau was significantly associated with spindle density, count, duration, and fast spindle density (Fig. 1) after controlling for age, sex, and ApoE4 genotype. CSF T-tau was not associated with slow spindle density, spindle power, or mean peak frequency. Fast spindles can be functionally differentiated from slow spindles by their greater tendency to nest within oscillations and promote memory processing [49]. In linear regression models where CSF T-tau, age, sex, and ApoE4 genotype where held constant as predictor variables, we observed that CSF T-tau explained 25% of the variance in N2 spindle count (F _(4, 49)_=3.77, *p* = 0.010), 45% of the variance in spindle duration (F_(4, 49)_=9.23, *p* < 0.001), and 41% of the variance in fast spindle density (F_(4, 49)_=7.90, p < 0.001) (Additional file 4: Table S3).

The cohort examined in this study contains significant overlap with subjects in which we found associations between measures of SWS and CSF Aβ_42_ (39/50 subjects) [12]. Consistent with our prior observations, CSF Aβ_42_ was inversely correlated with frontal SWA (r = − 0.312, *p* = 0.039 after correcting for age, sex, and ApoE4, Table 2*)*. In contrast, while we observed an unadjusted correlation between frontal SWA and T-tau, frontal SWA was not correlated with either T-tau or P-tau (r = − 0.291, *p* = 0.055 and r = − 0.191, *p* = 0.215, Table 2) when correcting for age, sex, and ApoE4.

### N2 spindle density is associated with neurocognitive measures

Both levels of tau in the brain and sleep spindle density have been associated with measures of cognition. In particular, sleep spindle density has shown to be correlated with the Bells Test, Connors Continuous Performance test, and Auditory Verbal Learning Test when completed the morning following sleep testing [50]. In the current study, neuropsychological testing was completed prior to the sleep study with variable duration between the administration of the tests and the in-lab sleep measurements. Across all subjects, we observed significant bivariate correlations between raw performance values on the digit symbol substitution test and fast spindle density, between Trails A raw performance values and both total and fast spindle density, and between Trails B raw performance values and slow spindle density. When scores on these tests were z-scored and normalized for age, sex, race, and years of education [40], we continued to observe significant correlations between Trails A performance and both total and fast spindle density, and between Trails B performance and slow spindle density (Additional file 5: Table S4). In all cases, higher spindle density was associated with better performance, except for slow spindle density, where greater slow spindle density was associated with longer times on Trails B.

## Discussion

Growing evidence suggests that disruptions in the sleep-wake cycle may increase AD risk prior to clinical symptoms. While attenuated SWS has been shown to be associated with elevated CSF Aβ [12, 13] as well as increased PET amyloid uptake [51], other changes in sleep oscillations have not been well characterized with respect to other AD biomarkers. In this study, we investigated the relationship between sleep spindles and CSF T-tau, P-tau and Aβ_42_ in a population of older cognitively normal adults. Spindle density in N2 sleep was significantly correlated with CSF T-tau, P-tau and Aβ_42_ both unadjusted and after adjustment for age, sex, and ApoE4. Nonetheless, because of the inter-correlations between CSF T-tau, P-tau and Aβ_42_, we posit that the association between Aβ_42_ and spindle density might be largely driven by the primary association of CSF tau with spindle density. This was supported by the loss of significant associations between CSF Aβ_42_ and spindle density when CSF T-tau or P-tau was included as a covariate.

We interpret the association of low spindle density with elevated CSF T-tau and P-tau as representative of increased AD risk. It is less clear that the association of low spindle density with elevated CSF Aβ_42_ is also reflective of increased AD risk, given that CSF Aβ_42_ is lower in bonafide AD dementia subjects when compared to age matched controls [52]. That said, how CSF Aβ_42_ changes over time, particularly in cognitively normal individuals who may be remote from developing symptoms remains a matter of some debate. For example, individuals with dominantly inherited AD have higher concentrations of CSF Aβ_42_ on average than controls 20 or more years from estimated symptom onset that then decline more rapidly [53, 54]. Additionally, recent data from the NYU, ADNI and NACC cohorts suggests an early preclinical stage, marked by CSF elevations in tau accompanied by elevations in CSF Aβ_42_ which were largely observed in younger age quartiles (between 45 and 70 years of age), indicating that elevated CSF Aβ_42_ might also be associated with very early preclinical AD risk [55]. When viewing CSF Aβ_42_ in isolation, we cannot rule out the possibility that low spindle density being associated with elevated Aβ_42_ might be protective. However, it is worth highlighting that spindle density was also significantly inversely correlated with CSF T-tau/Aβ_42_ and P-tau/Aβ_42_ which is meaningful because these CSF ratios have predicted decline from normal cognition in cognitively normal older adults over a 1–8 year follow-up period [56].

In addition to total spindle density, we also found evidence that spindle duration and fast spindle density during N2 sleep are significantly associated with CSF T-tau. Of note, a decrement in total spindle density and fast spindle density has also been observed in AD patients, who presumably have high brain tau load [57]. When evaluating other properties of sleep, we confirmed our prior observation that frontal SWA is associated with CSF Aβ_42_, however, SWA, SE, WASO and number of arousals during sleep were not correlated with CSF tau proteins in this sample.

This seeming specificity of CSF T-tau for spindles stands in potential contrast to recent findings suggesting that measures of subjective sleep quality by questionnaire were negatively correlated with both CSF T-tau/Aβ_42_ and P-tau/Aβ_42_ ratios [58]. This may be explained by differences in the use of subjective versus objective measures of sleep used in this study. Several other observations suggest that sleep disruptions from a variety of sources including insomnia [59], OSA [60–62] and arousals measured with actigraphy [18] increase risk for cognitive decline and development of AD. Our current results support further longitudinal investigation into whether spindle density is a sleep variable that also increases AD risk. Of note, spindle density did not correlate with general measures of sleep quality in these older subjects without documented sleep disorders, highlighting the utility of multidimensional and oscillation-specific analysis of sleep physiology such as the one used in this study.

While any correlation can represent an epiphenomenon, the observed associations between sleep spindles and CSF T-tau warrant thinking about possible mechanistic underpinnings that link the two. One possibility to consider is that accumulating tau negatively impacts the generation of spindles. Spindles are thought to largely evolve from a thalamo-cortical feedback loop involving thalamic reticular neuron inhibition of thalamic relay neurons. Tau is not thought to accumulate in thalamic reticular neurons, but tau in cortical neurons could affect this circuit. Tau in brainstem neurons projecting to thalamic reticular neurons could be involved [63–65], but would be expected to have more diffuse effects on sleep. In any such cases, reduced spindle density could serve as a biomarker of this pathological process [65].

The converse possibility is that sleep spindles reduce the production or increase the metabolism of tau. Animal studies have shown that neuronal activity can promote tau production [66, 67], however, spindles are an organized network pattern, and do not represent a simple decrease in overall network activity [19]. Additionally, sleep in general has been associated with immune function [68] and an increase in interstitial space in the brain [69], both of which constitute plausible mechanisms contributing toward reducing extracellular soluble tau in the brain. To date however, a specific role for sleep spindles in these processes has not been identified. Irrespective of a full understanding of the mechanism, the ability to manipulate sleep spindles with drugs [70] or with greater temporal precision using oscillating acoustic or other stimuli [71] establishes intriguing opportunities to test a causal role for sleep spindles in tau aggregation in human subjects.

Finally, spindle density and CSF tau levels could be associated by being common downstream consequences of an upstream process. For example, tau is released in response to axonal injuries (often from traumatic brain injury) at the time of injury and before frank evidence of neurodegeneration is present. Because we observed associations of sleep spindles with both CSF T-tau and P-tau, we cannot completely infer whether any association is more strongly tied to axonal injury versus formation of neurofibrillary tangles. In subjects with moderate to severe diffuse axonal injury, sleep spindle amplitude and peak frequency were significantly reduced acutely with subsequent return to baseline levels commensurate with functional improvement over 1 year [72]. Tauopathy is also observed in other neurodegenerative disorders including frontotemporal dementia, progressive supranuclear palsy (PSP), primary age related tauopathy and chronic traumatic encephalopathy. If there is a common upstream process that generally promotes tauopathy, reduced spindle density could be a corresponding consequence. In a study that included analysis of sleep spindles in subjects with PSP, spindle density was markedly reduced [73], supporting this idea.

Limitations of this work include the fact that the NPSG can itself alter sleep quality [74] in ways that vary between subjects. However, it should be noted that all subjects completed home monitoring for OSA screening prior to the in-lab NPSG that constituted the source of sleep data. Also, while we strived to perform LPs at a consistent time of day, the time interval between the LP and NPSG was variable and could constitute a source of variance. However, there is low intra-individual variability over 6 months to 2 years in CSF levels of T-tau, P-tau and Aβ_42_ [75]. Finally, it bears noting that when using a preclinical AD classification [76], our cohort contains a distribution of putative AD pathology, ranging from 48% without evidence for either CSF Aβ_42_ or tau to 8% that have presence of both CSF Aβ_42_ and tau above biomarker cutoffs. Therefore caution is warranted in interpreting the prognostic value of sleep spindle density, and studies tracking how sleep spindles change longitudinally along the progression of AD should be insightful.

## Conclusions

In conclusion, we report that spindles during N2 sleep are negatively correlated with CSF T-tau levels and to a lesser degree to CSF P-tau and CSF Aβ_42_. Further, sleep quality measures were not correlated with these CSF measures. These results indicate that poor sleep quality alone does not reduce spindle occurrence and is unlikely to be an explanatory factor in the observed associations between spindle density and levels of CSF tau. While reductions in NREM sleep oscillations, including SWS and spindles, may reflect a preclinical AD state, it remains unclear just how these attenuated oscillations are mechanistically linked to the cellular processes regulating Aβ and tau. Candidate mechanisms would include patterned activity-driven synthesis or degradation of such molecules or the coupling of such oscillations to neuro-immune function. Longitudinal and interventional studies to assess changes in sleep oscillations as well as AD biomarkers are needed.

## Additional files

Additional file 1: **Figure S1.** *Distribution of CSF biomarkers in this cohort.* Histograms of CSF Aβ_42_ (**A**), T-tau (**B**), P-tau_181_ (**C**) in our cohort of 50 cognitively normal elderly. **Figure S2.** *Sleep quality measures are not associated with either CSF T-tau at cross section*. Scatter plots of WASO (min.) (**A**), Sleep efficiency (%) (**B**), Arousal Index (#/min. of sleep) (**C**) or total sleep time (hours by habitual actigraphy, *n* = 39) (**D**) with CSF T-tau show no relationship at cross-section. (PDF 1529 kb)

Additional file 2: **Table S1.** Hierarchical linear regression examining CSF T-tau/Aβ_42_ ratio as a function of sleep measures (DOCX 15 kb)

Additional file 3: **Table S2.** Hierarchical linear regression examining CSF T-tau as a function of N2 spindle density with comorbidities (DOCX 15 kb)

Additional file 4: **Table S3.** Linear regression examining spindle properties as a function of CSF T-tau (DOCX 15 kb)

Additional file 5: **Table S4.** Correlations between neurocognitive tasks and spindle densities (DOCX 15 kb)

## Abbreviations

AHI: Apnea-Hypopnea Index
CDR: Clinical Dementia Rating
CPAP: Continuous positive airway pressure
CSF: Cerebrospinal fluid
DETOKS: Detection of K-complexes and sleep spindles
EEG: Electroencephalographic
EMG: Electromyographic
EOG: Electrooculographic
ESS: Epworth Sleepiness Scale
FFT: Fast Fourier transform
LP: Lumbar puncture
MCI: Mild cognitive impairment
MMSE: Mini-Mental State Examination
N1: Non-REM stage 1 sleep
N2: Non-REM stage 2 sleep
N3: Non-REM stage 3 sleep
NFT: Neurofibrillary tangles
NPSG: Nocturnal polysomnography
OSA: Obstructive sleep apnea
REM: Rapid Eye Movement
SE: Sleep efficiency
SWA: Slow wave activity
SWS: Slow wave sleep
TST: Total sleep time
W: Wake
WASO: Wake after sleep onset
