# Supplementary figures and images for: Sleep oscillation-specific associations with Alzheimer’s disease CSF biomarkers: novel roles for sleep spindles and tau
Source: Mol Neurodegener. 2019 Feb 21;14:10. doi: 10.1186/s13024-019-0309-5 (PMC6385427; doi:10.1186/s13024-019-0309-5)

Figure S1

A.

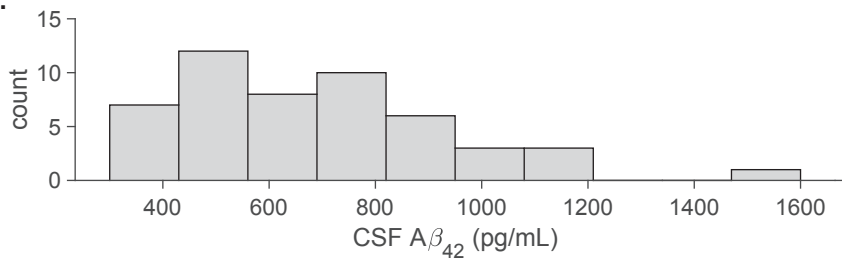

B.

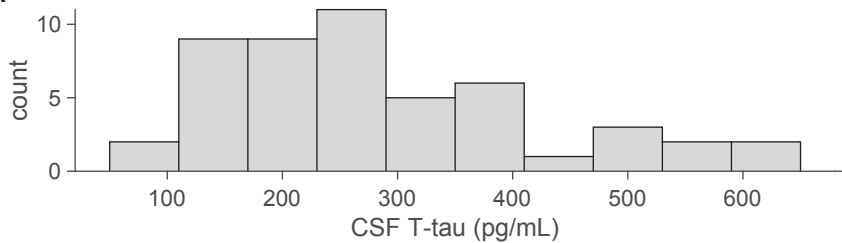

C.

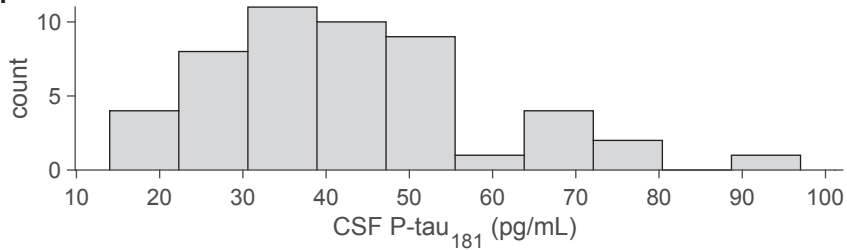

Figure S2

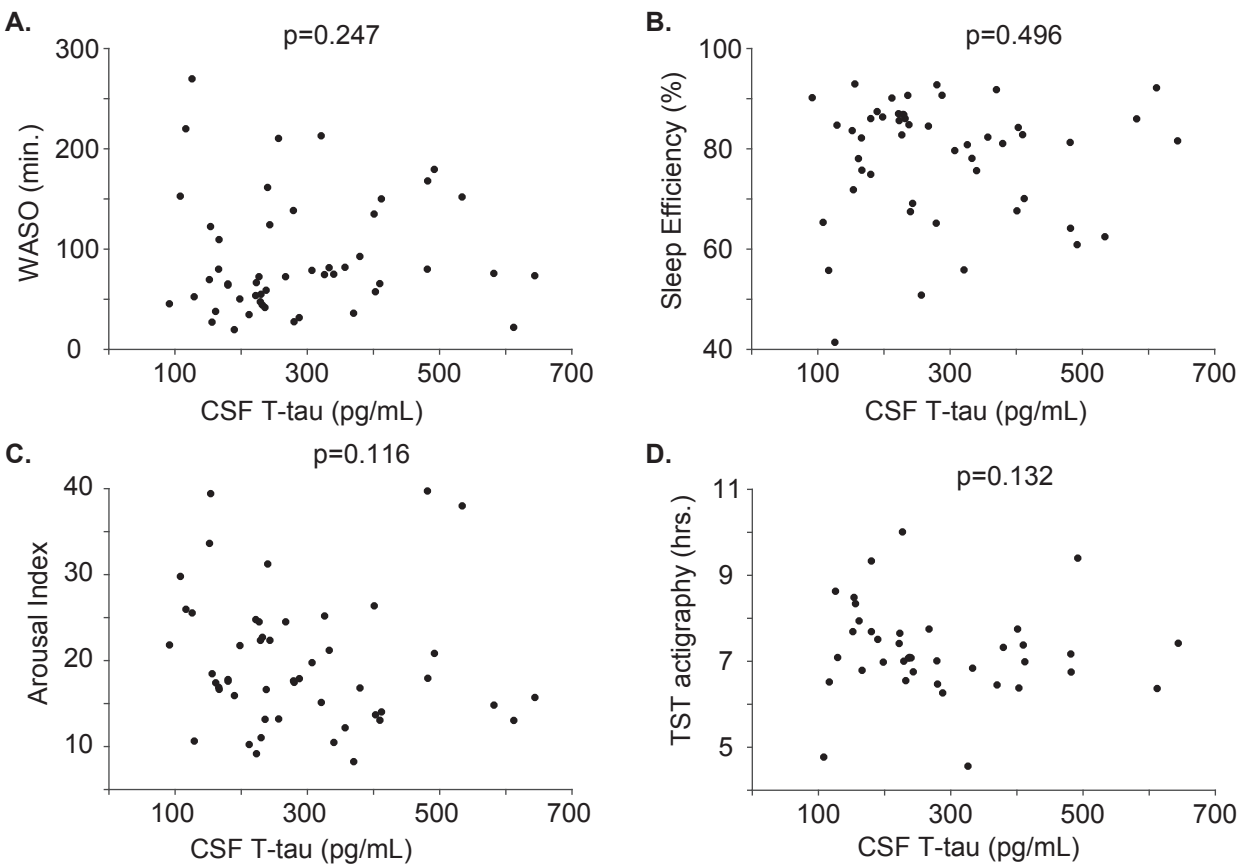

Supplement: Supplementary file 1 — Figure S1. Distribution of CSF biomarkers in this cohort. Histograms of CSF Aβ42 (A), T-tau (B), P-tau181 (C) in our cohort of 50 cognitively normal elderly. Figure S2. Sleep quality measures are not associated with either CSF T-tau at cross section. Scatter plots of WASO (min.) (A), Sleep efficiency (%) (B), Arousal Index (#/min. of sleep) (C) or total sleep time (hours by habitual actigraphy, n = 39) (D) with CSF T-tau show no relationship at cross-section. (PDF 1529 kb) [file 13024_2019_309_MOESM1_ESM.pdf]
