# Supplementary material for: Sleep oscillation-specific associations with Alzheimer’s disease CSF biomarkers: novel roles for sleep spindles and tau
Source: Mol Neurodegener. 2019 Feb 21;14:10. doi: 10.1186/s13024-019-0309-5 (PMC6385427; doi:10.1186/s13024-019-0309-5)
Supplement: Supplementary file 2 — Table S1. Hierarchical linear regression examining CSF T-tau/Aβ42 ratio as a function of sleep measures (DOCX 15 kb) [file 13024_2019_309_MOESM2_ESM.docx]

# Table S1: Hierarchical linear regression examining CSF T-tau/Aβ_42_ ratio as a function of sleep measures

| Model ^a^ | Predictors | β | 95% CI | p ^c^ | R^2^ | ΔR^2^ |
| --- | --- | --- | --- | --- | --- | --- |
| Model 6: age, sex and ApoE4 status only | Age | 0.236 | -0.001, 0.005 | 0.130 | 0.310 | NA |
|  | Sex | 0.112 | -0.026,0.055 | 0.464 |  |  |
|  | ApoE4 | 0.491 | 0.026, 0.107 | **0.002** |  |  |
| Models 7 ^b^: age, sex and ApoE4 status plus one sleep variable | N2 spindle density | -0.284 | -0.08, 0.004 | 0.072 | 0.379 | 0.070 |
|  | SWA | -0.036 | -0.045, 0.037 | 0.835 | 0.311 | 0.001 |
|  | WASO | -0.160 | -0.050, 0.017 | 0.330 | 0.331 | 0.021 |
|  | SE | 0.127 | -0.074, 0.174 | 0.421 | 0.324 | 0.014 |
|  | AHI4% | -0.231 | -0.026, 0.003 | 0.118 | 0.363 | 0.053 |
|  | AHI-all | -0.203 | -0.051, 0.011 | 0.207 | 0.344 | 0.035 |
|  | TST in-lab | -0.063 | -0.025, 0.017 | 0.690 | 0.313 | 0.004 |
|  | TST actigraphy | -0.006 | -0.021, 0.020 | 0.971 | 0.310 | 0.000 |

a. dependent variable: T-tau/Aβ_42_.

b. change from model which only includes covariates age, sex, and ApoE4.

c. significance level for each predictor.

Model 7 did not have a significant change in the amount of variance explained.
