# Supplementary material for: Sleep oscillation-specific associations with Alzheimer’s disease CSF biomarkers: novel roles for sleep spindles and tau
Source: Mol Neurodegener. 2019 Feb 21;14:10. doi: 10.1186/s13024-019-0309-5 (PMC6385427; doi:10.1186/s13024-019-0309-5)
Supplement: Supplementary file 3 — Table S2. Hierarchical linear regression examining CSF T-tau as a function of N2 spindle density with comorbidities (DOCX 15 kb) [file 13024_2019_309_MOESM3_ESM.docx]

# Table S2: Hierarchical linear regression examining CSF T-tau as a function of N2 spindle density with comorbidities

| Model ^a^ | Predictors | β | 95% CI | p ^c^ | R^2^ | ΔR^2^ |
| --- | --- | --- | --- | --- | --- | --- |
| Model 8: age, sex, ApoE4 status and N2 density | Age | -0.125 | -0.025, 0.010 | 0.380 | 0.400 | NA |
|  | Sex | -0.256 | -0.486, 0.014 | 0.063 |  |  |
|  | ApoE4 | 0.461 | 0.193, 0.705 | **0.001** |  |  |
|  | N2 Spindle density | -0.487 | -0.776, -0.214 | **0.001** |  |  |
| Model 9 ^b^: age, sex, ApoE4 status and N2 density plus comorbidities | Hypertension | -0.027 | -0.298, 0.246 | 0.848 | 0.465 | 0.064 |
|  | Cardiovascular disease | 0.137 | -0.500, 1.325 | 0.364 |  |  |
|  | Diabetes | 0.243 | -0.151, 1.612 | 0.101 |  |  |
|  | Thyroid disorders | -0.014 | 0.353, 0.322 | 0.924 |  |  |

a. dependent variable: T-tau.

b. change from model which only includes covariates age, sex, and ApoE4.

c. significance level for each predictor.

Model 9 did not have a significant change in the amount of variance explained.
