# Supplementary material for: Sleep oscillation-specific associations with Alzheimer’s disease CSF biomarkers: novel roles for sleep spindles and tau
Source: Mol Neurodegener. 2019 Feb 21;14:10. doi: 10.1186/s13024-019-0309-5 (PMC6385427; doi:10.1186/s13024-019-0309-5)
Supplement: Supplementary file 4 — Table S3. Linear regression examining spindle properties as a function of CSF T-tau (DOCX 15 kb) [file 13024_2019_309_MOESM4_ESM.docx]

# Table S3: Linear regression examining spindle properties as a function of CSF T-tau

| Dependent variable | Predictors | R^2^ | β | 95% CI | p ^a^ |
| --- | --- | --- | --- | --- | --- |
| N2 count | Age | 0.251 | -0.233 | -0.373, 0.026 | 0.087 |
|  | Sex |  | -0.071 | -3.866, 2.252 | 0.598 |
|  | ApoE4 |  | 0.238 | -0.399, 6.119 | 0.084 |
|  | T-tau |  | -0.419 | -8.444, -1.743 | **0.004** |
| Duration | Age | 0.451 | -0.200 | -0.012, 0.001 | 0.086 |
|  | Sex |  | -0.022 | -0.110, 0.091 | 0.847 |
|  | ApoE4 |  | 0.320 | 0.040, 0.255 | **0.008** |
|  | T-tau |  | -0.621 | -0.400, -0.180 | **<0.001** |
| Fast spindle density | Age | 0.413 | -0.264 | -0.027, -0.001 | **0.030** |
|  | Sex |  | -0.084 | -0.268, 0.128 | 0.478 |
|  | ApoE4 |  | 0.417 | 0.155, 0.577 | **0.001** |
|  | T-tau |  | -0.505 | -0.666, -0.232 | **<0.001** |

a. significance level for each predictor.
