# Supplementary material for: Sleep oscillation-specific associations with Alzheimer’s disease CSF biomarkers: novel roles for sleep spindles and tau
Source: Mol Neurodegener. 2019 Feb 21;14:10. doi: 10.1186/s13024-019-0309-5 (PMC6385427; doi:10.1186/s13024-019-0309-5)
Supplement: Supplementary file 5 — Table S4. Correlations between neurocognitive tasks and spindle densities (DOCX 15 kb) [file 13024_2019_309_MOESM5_ESM.docx]

**Table S4: Correlations between neurocognitive tasks and spindle densities**

|  | Trails A | | Trails B | | DSST | |
| --- | --- | --- | --- | --- | --- | --- |
|  | rho (p)^a^ | rho (p)^b^ | rho (p)^a^ | rho (p)^b^ | rho (p)^a^ | rho (p)^b^ |
| Total Spindle density N2 sleep | **-0.422 (0.007)** | **0.321 (0.043)** | 0.064 (0.661) | -0.156 (0.285) | 0.253 (0.079) | 0.142 (0.330) |
| Fast Spindle density N2 sleep | **-0.555 (0.000)** | **0.390 (0.013)** | -0.196 (0.177) | 0.063 (0.666) | **0.360 (0.011)** | 0.218 (0.133) |
| Slow Spindle density N2 sleep | 0.033 (0.840) | -0.018 (0.911) | **0.361 (0.011)** | **-0.306 (0.032)** | -0.037 (0.803) | -0.032 (0.830) |

a. Spearman's correlations of Neurocognitive tasks (unadjusted) to spindle density (unadjusted)

b. Spearman's correlations of Neurocognitive tasks (robust Z-transform per De Santi et al. 2008) to spindle density (unadjusted)

DSST = digit symbol substitution test
